# Developing the next generation of graphene-based platforms for cancer therapeutics: The potential role of reactive oxygen species

**DOI:** 10.1016/j.redox.2017.11.018

**Published:** 2017-11-22

**Authors:** Tanveer A. Tabish, Shaowei Zhang, Paul G. Winyard

**Affiliations:** a College of Engineering, Mathematics and Physical Sciences, University of Exeter, Stocker Road, Exeter EX4 4QF, United Kingdom; b Institute of Biomedical and Clinical Science, University of Exeter Medical School, St Luke's Campus, Exeter EX1 2LU, United Kingdom

**Keywords:** PDT, photodynamic therapy, ROS, reactive oxygen species, GO, graphene oxide, HIF-1ɑ, hypoxia-inducible factor-1 alpha, NF-ϰB-NF kappa B, nuclear factor kappa-light-chain-enhancer of activated B cells, PTEN, phosphatase and tensin homolog deleted on chromosome 10, AP-1, activator protein-1, Hh, hedgehog, STAT3, signal transducer and activator of transcription 3, Rb, retinoblastoma, Nrf2, nuclear factor erythroid-derived 2-like 2, Sp1, specificity protein 1, PPa, Pyropheophorbide-a, mAb, monoclonal antibody, Graphene, Reactive oxygen species, Singlet oxygen, Theranostics, Photodynamic therapy, Bioimaging

## Abstract

Graphene has a promising future in applications such as disease diagnosis, cancer therapy, drug/gene delivery, bio-imaging and antibacterial approaches owing to graphene's unique physical, chemical and mechanical properties alongside minimal toxicity to normal cells, and photo-stability. However, these unique features and bioavailability of graphene are fraught with uncertainties and concerns for environmental and occupational exposure. Changes in the physicochemical properties of graphene affect biological responses including reactive oxygen species (ROS) production. Lower production of ROS by currently available theranostic agents, e.g. magnetic nanoparticles, carbon nanotubes, gold nanostructures or polymeric nanoparticles, restricts their clinical application in cancer therapy. Oxidative stress induced by graphene accumulated in living organs is due to acellular factors which may affect physiological interactions between graphene and target tissues and cells. Acellular factors include particle size, shape, surface charge, surface containing functional groups, and light activation. Cellular responses such as mitochondrial respiration, graphene-cell interactions and pH of the medium are also determinants of ROS production. The mechanisms of ROS production by graphene and the role of ROS for cancer treatment, are poorly understood. The aim of this review is to set the theoretical basis for further research in developing graphene-based theranostic platforms.

## 1 Introduction

**Fig. 1:**
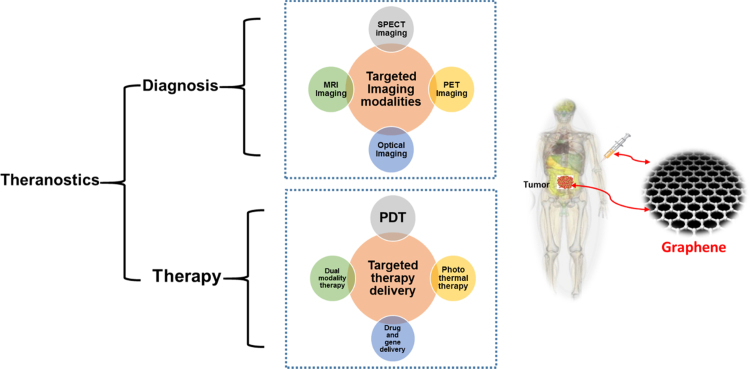
Schematic representation of a typical theranostic platform for the combined use of a range of imaging and therapeutic approaches. Imaging modalities include: ultrasonography, positron electron tomography, fluorescence imaging, magnetic resonance imaging and single-photon emission computed tomography. Therapeutic approaches include: drug delivery, photothermal therapy, photodynamic therapy, or a combination of two therapies. Based on its unique properties, graphene can be employed as a theranostic agent that combines the capabilities of diverse imaging and therapeutic modalities to target tumors.

Cancer is one of the leading causes of morbidity and mortality worldwide, with more than 14 million new cases and 8.8 million deaths in 2012 [1]. Globally, cancer accounts for nearly one of every six deaths. Cancer elicits a significant economic cost. The total annual economic cost of cancer in 2010 was estimated at approximately US$ 1.16 trillion [2]. Conventional therapeutic options including chemotherapy and radiation therapy are most commonly used in the treatment of cancer. However, these modalities yield low success rates and have profound adverse side effects on patients' physical and mental health [3]. Therefore less invasive, and more effectively targeted, treatments need to be developed for palliative care and improvement of quality of life. Novel regimes for simultaneous diagnosis and therapy, known as theranostics, have changed the cancer treatment algorithm by the combination of bio-imaging with site-specific and site-selective targeting of tumors, without damaging normal cells [4]. A schematic representation of the components of a typical theranostic platform is given in Fig. 1.

The two key components of this theranostic platform are: first, targeted diagnostic imaging modalities and, secondly, targeted delivery of therapies such as photodynamic therapy (PDT). An excellent review of targeted diagnostic imaging has recently been contributed by Cope et al. (2016) [5]. PDT has evolved into a practical, effective and systematic theranostic option comprising of the multiple-exposure, guided, non-invasive, treatment of tumors in combination with real-time detection and tracking of malignant tissue by fluorescence imaging. The basis of PDT is that light is utilized to activate a non-toxic photosensitizer, leading to the generation and localization of highly toxic reactive oxygen species (ROS) at the targeted site of cancerous tissue. PDT offers several advantages over traditional treatment options, typically including low toxicity of the photosensitizer in the absence of light interaction/irradiation, better efficacy, low side effects, selective and specific accumulation, and deep penetration of photosensitizer into the tumors [6]. Nevertheless, the mechanism of the selective and specific killing of tumor cells by ROS remains unclear. A better understanding of this phenomenon will empower patients and clinicians with a greater confidence in this treatment option.

A key feature of PDT is to exploit the light source for selective activation of the photosensitizer within the tumor cells. A light source of appropriate wavelength (visible or near-infrared) is used to activate a photosensitizer that generates and releases ROS, for the selective killing of tumors [7]. The photo-activation of the photosensitizer initially enables its excitation to a triplet state through a short-lived intermediate called the ‘singlet state’. The electron and energy transfer to the surrounding free oxygen produces ROS, including singlet oxygen, the superoxide anion radical, the hydroxyl radical, and hydrogen peroxide. Highly toxic ROS cause tumor cell death by oxidative stress.

Historically, the development of photosensitizers has resulted in three eminent generations of photosensitizer types. The first generation is porphyrins [8]. The clinical limitations of porphyrins are poor selectivity, poor photosensitivity, a low clearance rate, and a low light penetration within tumors. The second generation of photosensitizers - including chlorins, porphyrinoids and transition metal complexes - also has several problems such as: high hydrophobicity, poor tumor selectivity, complex surface chemistry, and aggregation in aqueous media. The third generation includes biomolecule conjugates and covalently attached peptides [8]. The selection of biomolecules is critical for their clinical efficacy because of the selective targeting capability, the structural and photochemical properties of these conjugates, and the degree of receptor expression in the targeted tumors.

**Fig. 2:**
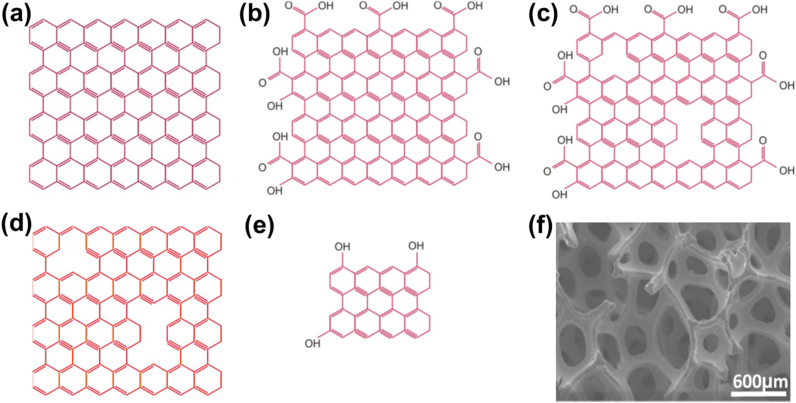
Summary of structural models of various derivatives of graphene. (a) Graphene, (b) graphene oxide (GO), (c) reduced graphene oxide, (d) porous graphene, (e) graphene quantum dots and (f) three dimensional graphene foam. Graphene is a sp^2^ hybridized model of carbon atoms in a repeated manner, forming a regular lattice structure (as shown in panel a), while GO and reduced GO have functional groups and defects in their basal planes (panels b and c). The physicochemical properties and structures of different graphene variants depend on the fabrication method and conditions. The presence of both defects and functional groups provides potential advantages for the efficient utilization of graphene variants in the production of ROS. The chemical exfoliation method is thought to be an efficient route for synthesizing graphene on a large scale and at low cost. Porous graphene is a graphene sheet that is missing carbon atoms from its plane. The various forms of porous graphene provide fascinating materials for biological applications owing to their high specific surface areas, hydrophobic nature and biocompatibility. Graphene nanopores usually have pore sizes of 1–30 nm. Pores and vacancies can clearly be seen in the porous graphene sheet, as represented in panel (d). Graphene quantum dots are luminescent nanocrystals having a size less than 50 nm. These have attractive properties and potential applications in cancer diagnosis and treatment. Water soluble graphene quantum dots, shown in panel (e), have functional groups (C–OH, C=O, C–O–C, C–H) on their surface. Three-dimensional graphene networks in the form of a foam, sponge or aerogel have recently been assembled from individual graphene sheets using chemical vapour deposition templated methods, which also preserve the unique properties of individual graphene sheets. [Panel (f) is adapted from [12], with permission of MDPI Publishing Group, Copyright 2015].

Recently, novel photosensitizers have been fabricated to improve the selective tissue penetration of incident light, and to improve the clinical efficacy of PDT. Among such novel developments, graphene has also recently been fabricated and utilized as a photosensitizer and theranostic agent [9]. Graphene is a two-dimensional single-layer-thin material with sp^2^-bonded carbon atoms packed into a honeycomb lattice. This material has gained significant attention in many disciplines of life science, owing to its electronic, optical and structural properties. Graphene has been applied as a drug carrier in chemotherapy, and as a photosensitizer for photothermal therapy and PDT. A graphene nanohybrid showed improved anticancer PDT effects compared to the conventional photosensitizers [10]. Graphene has significant potential for use in theranostic agents due to many fascinating properties, such as a high specific surface area, appropriate energy and/or electron transfer features, a high fluorescence quantum yield, π−π stacking, good water dispersibility, good biocompatibility, enhanced drug-loading efficiency, selective tumor uptake, minimal side effects and a high yield of ROS production. Graphene has a variety of derivatives including graphene oxide (GO), reduced graphene oxide, graphene quantum dots, graphene nanoribbons, three dimensional graphene foam and graphene nanopores. The structural models of several graphene derivatives are shown in Fig. 2. GO is a highly efficient long-range quencher for various fluorescence processes [11].

The therapeutic responses of different derivatives of graphene such as GO and graphene quantum dots revealed them as promising treatment agents and showed the possibility of exploiting ROS in cancer treatment. A better understanding of the role of ROS in the therapeutic mode of action of graphene, in cancer treatment, will facilitate the development of improved graphene-based theranostic platforms.

## 2 Toxic potential of graphene family nanomaterials

**Fig. 3:**
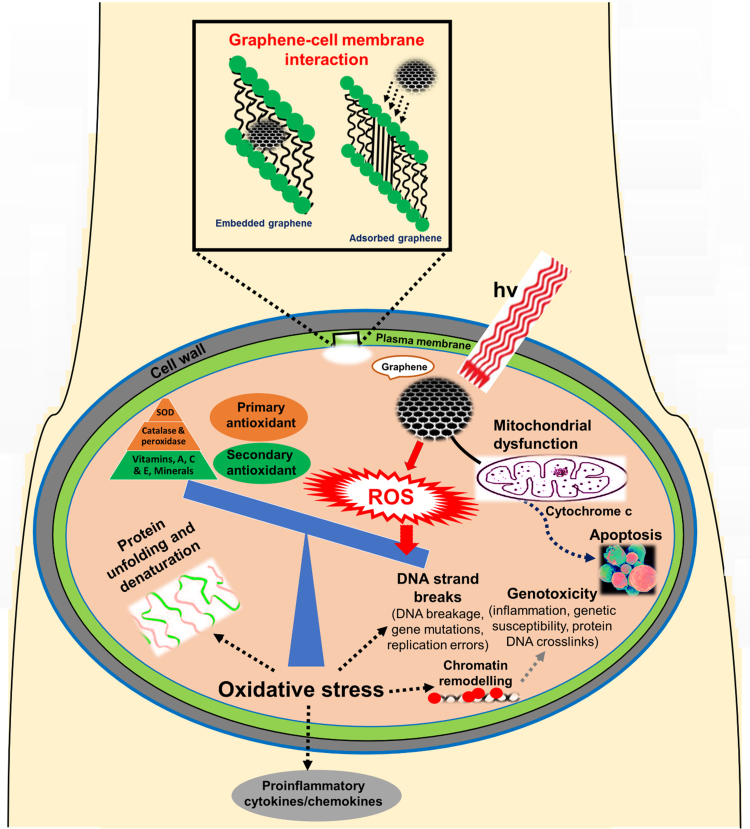
Schematic illustration of the potential mechanisms by which reactive oxygen species (ROS) are associated with the cellular toxicity of graphene. Graphene may affect biological behavior at the cellular, subcellular, protein and gene levels. The toxicity of graphene depends on its physicochemical interactions and its accumulation in specific organs. Uptake of graphene into specific organs also affects cell function as a result of cellular changes within the organs. The deposition, distribution and clearance of graphene after entering into a living system is a major knowledge gap in understanding the toxicity of graphene. Graphene circulating in the bloodstream is internalized into cells through the plasma membrane. The plasma membrane is a selectively permeable membrane that transfers materials such as ions and nano-sized proteins. Graphene (depending on its size, shape, and surface chemistry) enters the cell via different pathways such as clathrin/caveolar-mediated endocytosis, phagocytosis, macropinocytosis, and pinocytosis and exits the cell via the pathways of lysosome secretion, vesicle-related secretion, and non-vesicle-related secretion. The nature of plasma membrane interaction with graphene determines the fate of graphene in a wide range of potential applications with high biocompatibility, including drug- and gene-delivery, photothermal and photodynamic therapy. This interaction may lead to the possibility of events such as adsorption or incorporation of graphene onto the surfaces of cells. Furthermore, the entrapped biomolecules on the surface of graphene, when graphene is present within the extracellular matrix, may influence the tertiary structure of a protein - resulting in the formation of a protein-graphene interface and malfunction. The extracellular mechanisms causing the accumulation of graphene in the extracellular matrix and the subsequent effects of graphene on the extracellular matrix remain undefined. Graphene-induced ROS may cause oxidative stress, loss of cell function, mitochondrial damage, initiation of lipid peroxidation, covalent chemical modifications of nucleic acids, DNA-strand breaks, induction of gene expression via the activation of transcription factors, and modulation of inflammation via signal transduction - leading to toxicity, cell death and genotoxicity. The specific minerals in the secondary antioxidants are being referred to selenium, zinc, molybdenum, iron and copper. The antioxidant defence system is overwhelmed by high levels of ROS, leading to oxidative stress, inflammation and toxicity. One potential way to minimize the toxicity of graphene is to functionalize the graphene with biodegradable agents.

ROS generation by nanoparticles has been considered as the primary source of their toxicity [13]. Potential adverse effects of ROS include the downregulation of defensive systems to disrupt the structure and function of normal cells. ROS cause damage to cellular components such as proteins, DNA and lipids, resulting in the release of inflammatory cytokines and chemokines. ROS generation by graphene is dependent on several factors that strongly define the extent of graphene-induced toxicity, such as: size and shape, particle surface, surface charges, surface-associated chemical groups, solubility and dispersion, ions released from graphene, photo-activation, aggregation, mode of interaction with cells, the presence of inflammation in tissues, and the pH of the system. In addition, the conditions of experiments in which graphene is administered, either in vivo or in vitro, affect the interactions between graphene and targeted cells. Such conditions include the time of exposure, dose, and (in the case of in vitro experiments) the cell type and the criterion used for examining cell viability. For in vivo models, the method of administration is also of course crucial [9]. Graphene can cause an inflammatory response that produces relatively large amounts of free radicals such as hydroxyl radicals [14]. GO at a low concentration (< 4 μg/ml) resulted in a perturbation of mitochondrial structure and function in Hep G2 cells, as measured by a decrease in the mitochondrial membrane potential and the dysregulation of mitochondrial Ca^2+^ homeostasis [15], while higher concentrations of graphene quantum dots (< 200 μg/ml) also caused decreases in the mitochondrial membrane potential by increased ROS generation, in association with apoptotic and autophagic cell deaths, with an increase in the expression caspase 3, caspase 9, beclin 1, and microtubule-associated protein 1A/1B-light chain 3 [16]. Apoptosis and autophagy are two key modes of cancer cell death, in addition to necrosis. Apoptosis is a widely studied form of cell death and mainly originates through the activation of death receptors (extrinsic pathway) or through mitochondrial permeabilization (intrinsic pathway). ROS play a key role in both the extrinsic and intrinsic pathways of apoptosis, as initiators and in enabling signaling events. The apoptosis-inducing ligand, Fas, produces ROS in the extrinsic pathway of the apoptotic process [17]. Activation of the extrinsic pathway requires an inflammatory response to tissue injury and may cause a delay in intrinsic pathway initiation that responds immediately to calcium and ROS. Oxidative stress may be associated with the intracellular accumulation of ROS. Moreover, increased intracellular ROS levels, with associated increases in apoptosis, were detected in murine RAW 264.7 macrophages exposed to graphene (20–100 µg/ml) [18]. Chang et al. [19] reported a concentration-dependent toxicity of GO on A549 cells in vitro, a concentration of 200 µg/ml causing a dose-dependent oxidative stress in cells and inducing a loss of cell viability. However it was also found that a low concentration of GO (10 μg/ml) did not enter A549 cells and had no obvious toxicity. The higher concentration of GO (200 µg/ml) caused oxidative stress and induced a slight loss of cell viability. Oxidative stress as a result of graphene-cell interactions may cause cell mutagenesis, carcinogenesis and ageing [20]. Graphene may cause mitochondrial toxicity that includes changes in mitochondrial calcium levels and depletion of the mitochondrial membrane potential. Graphene subsequently triggers apoptosis by the activation of mitochondrial pathways, namely the mitogen-activated protein kinases (MAPKs) and transforming growth factor-β (TGF-beta)-related signaling pathways. Graphene has the potential to adsorb aromatic amino acids by π-π stacking [21]. Recent in vivo and in vitro studies [12], [14], [15], [16], [17], [18], [19], [20], [22], [23] have shown the role of ROS in mediating the toxicity of graphene. A schematic illustration of the potential ROS-mediated mechanisms manifested by graphene in the cell is shown in Fig. 3.

## 3 ROS in cancer theranostics

**Fig. 4:**
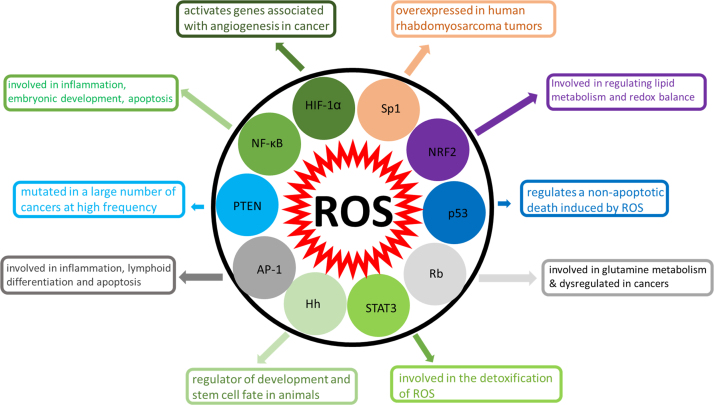
Cell signaling and molecular targets of ROS in cancer. ROS may induce both transcriptional factors/activators and genes associated with tumor suppression: HIF-1α (hypoxia-inducible factor-1 alpha); NF-κB (nuclear factor kappa-light-chain-enhancer of activated B cells); PTEN (phosphatase and tensin homolog deleted on chromosome 10); AP-1 (activator protein-1); Hh (hedgehog protein); STAT3 (signal transducer and activator of transcription 3); Rb (retinoblastoma protein); Nrf2 (nuclear factor (erythroid-derived 2)-like 2); Sp1 (specificity protein 1). NF-κB and AP-1 are transcription factors that play key roles in the expression of many genes involved in inflammation as well as many other significant events such as embryonic development, lymphoid differentiation and apoptosis. HIF-1α plays an essential role in embryonic vascularization and tumor angiogenesis. Nrf2, a redox-sensitive transcription factor, regulates genes which bind antioxidant response elements in DNA. PTEN is a tumor suppressor gene, which is deleted or mutated at high frequency in a large number of cancers. Rb protein is a tumor suppressor gene which controls cell cycle progression. Sp1 is a transcription factor which contributes to overexpression of MDM2 (mouse double minute 2 homolog) in rhabdomyosarcoma tumors. Stat 3 is a transcription factor which plays an important role in cell growth and apoptosis. ROS-mediated signaling through activation of these transcription factors controls the expression of genes involved in inflammation, metastasis, cell proliferation and tumor angiogenesis, as well as tumor cell death or survival.

The proof-of-concept investigations of graphene in cancer theranostics are still at a preclinical stage. An early report on GO as theranostic agent was published by Cho and his group [24]. They synthesized a GO-based photosensitizer with a redox-responsive disulfide linker which was activated by glutathione. This photosensitizer exhibited a remarkable fluorescence emission and singlet oxygen generation in the presence of glutathione as a reducing agent. There was efficient cellular internalization and preferential accumulation of the photosensitizer inside cancer cells, and glutathione was then able to cleave the disulfide linkers. Cho et al. demonstrated in vitro cellular uptake and fluorescence activation of the photosensitizer, but they did not report the role of ROS in phototoxicity towards A549 cells. As mentioned earlier, ascertaining the type of ROS produced, the nature of intracellular ROS signaling, ROS localization, and cancer cell-specific ROS-sensing mechanisms are the most important challenges in relation to understanding the role of the ROS in cell killing by graphene. The molecular targets of ROS in cancer are shown in Fig. 4. ROS may induce both transcription factors/activators and genes associated with tumor suppression [25].

Cancer cells possess an inherent nature of survival and re-growth [26]. Thus, the effectiveness of a therapy depends on the selective and specific targeting of tumors without producing chronic, severe, harm to vital organs and normal cells. Caspase activation by the intrinsic pathway leads to the release of cytochrome c, a family of proteins known as “inhibitors of apoptosis proteins” (IAP), and endonuclease G. Release of these factors leads to disintegration of the mitochondrial membrane to form the mitochondrial permeability transition pore complex. Cao et al. [27] prepared a multifunctional theranostic agent based on porphyrin-conjugated polyethylene glycol-functionalized graphene quantum dots. These functionalized graphene quantum dots demonstrated a clear discrimination (as observed by the use of a cell imaging label and intracellular micro RNA detection) of cancer cells from somatic cells. The functionalized graphene quantum dots also exhibited a high yield of singlet oxygen (quantum yield up to 1.08) with 28.58% photothermal conversion efficiency. Apoptotic events and cell membrane destruction were observed in A549 cells exposed to these porphyrin-conjugated polyethylene glycol-functionalized graphene quantum dots. However, porphyrin-based functionalized graphene quantum dots revealed a slightly slower ^1^O_2_ production rate compared with porphyrin alone. Wei et al. [28] prepared a nanodrug pyropheophorbide-a-nano GO-monoclonal antibody conjugate, within which the monoclonal antibody was directed against integrin α_v_β_3_ as a mechanism for tumor targeting. The authors demonstrated that the phototoxicity of GO-bound pyropheophorbide can be switched on and off in both organic and aqueous environments after the conjugation of pyropheophorbide with polyethylene-glycol. The functionalized GO efficiently targets the cancer cells’ surface ligand (i.e. integrin α_v_β_3_). Once endocytozed by the cells, and having then escaped from lysosomes, the functionalized GO subsequently moves to the mitochondria. The two-fold on/off switching of this functionalized GO considerably increases the intrinsic pathway of apoptosis.

**Fig. 5:**
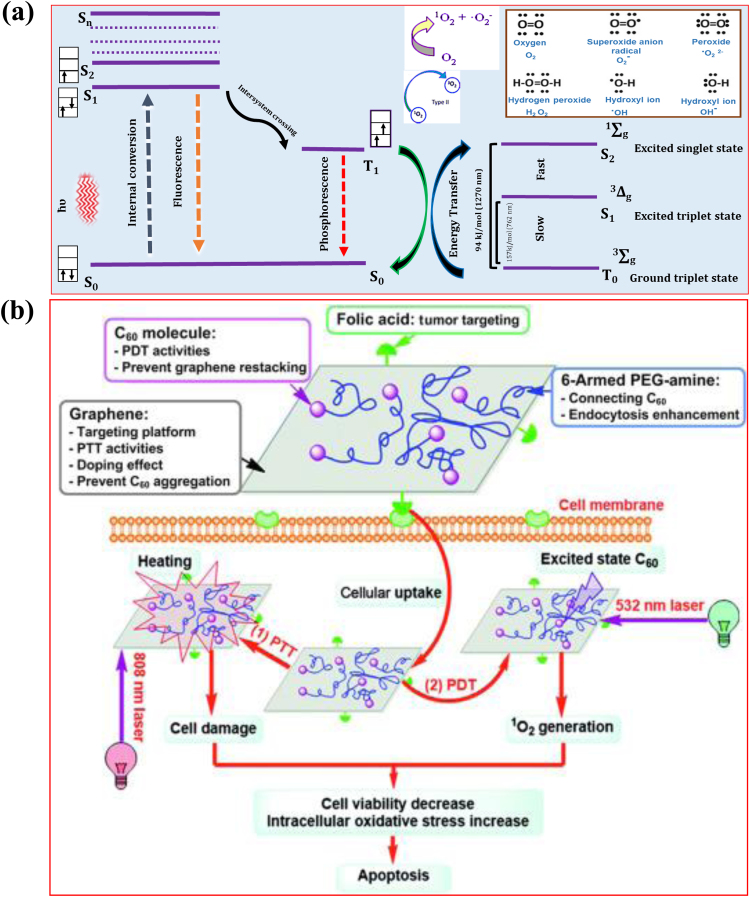
Schematic representations of the mechanism involved in singlet oxygen production leading to programmed cell death induced by combined photodynamic and photothermal therapies using a graphene nanocomposite photosensitizer. Photodynamic therapy (PDT) refers to the use of a non-toxic compound called a photosensitizer and a special laser light to kill cancer cells, while photothermal therapy (PTT) uses the heat generated from the absorbed optical energy by light-absorbing nanoparticles embedded within tumors to ablate tumor cells. Panel **(a)** shows a schematic illustration of the mechanisms of singlet oxygen (^1^O_2_) generation by a photosensitizer, in the form of a Jablonski diagram representing the electronic states of a photosensitizer after light absorption, followed by energy transfer to an oxygen molecule to generate ^1^O_2_. The photosensitizer displays intersystem crossing to the triplet state when the photosensitizer is excited to the singlet state. The electronic states are shown in the diagram. **Internal conversion:** transitions between states of similar electronic spin, where the electronic states are singlet and triplet. **Fluorescence:** the emitted photon has energy resembling the energy difference between the initial and final states of the photosensitizer. The emitting and final states have similar electronic spin states, either singlet or triplet. **Intersystem crossing:** the change of electronic spin in the excited state, from singlet to triplet. **Phosphorescence:** the emitted photon has energy resembling the energy difference between the initial and final states of the photosensitizer. The emitting and final states have different electronic spin states, such as one in the singlet state and the other in the triplet state. Panel **(b)** is a schematic illustration of the mechanism of cancer cell killing induced by a functionalized hybrid of folic acid (FA), polyethylene glycol (PEG) and C_60_ (a spherical fullerene molecule with the formula C_60_ called buckminsterfullerene) non-covalently conjugated to GO for synergistic combined photothermal therapy and photodynamic therapy. Thus, the functionalized hybrid consists of FA-GO-PEG/C_60_ [FA (cancer targeting moiety) and C_60_ (photosensitizer) conjugated to PEGylated graphene oxide]. Functionalized GO was exposed to light sources with wavelengths of 532 and 808 nm for enhanced cellular uptake of C_60_ in cancer cells. The GO nanocomposite showed effective cell apoptosis and death and exhibited a synergistic effect of combined photodynamic and photothermal therapies. [Panel (b) is adapted from [10], with permission of the Royal Society of Chemistry, Inc., Copyright 2015].

H_2_O_2_-induced apoptosis usually occurs in lymphoma cells via activation of cysteine proteases such as caspase-3 [29]. H_2_O_2_ is a precursor of highly reactive hydroxyl radicals, while H_2_O_2_ itself has relatively low reactivity. H_2_O_2_ produced by nanoparticles has shown great potential to initiate apoptosis in the cells of osteosarcomas, and in breast, bladder, and lung cancer cells. He et al. [30] reported a nanoagent based on iron hydroxide/oxide-modified GO and showed a higher generation of superoxide anion radicals under near-infrared light irradiation, compared with GO alone. In respect of this composite, it was proved that near-infrared light irradiation promoted electron transfer from GO to Fe(III) (endogenously present within the cells) and accelerated the formation of superoxide radicals. H_2_O_2_ (formed from superoxide) then reacted with Fe(II) and gave an improved yield of hydroxyl radicals. Excessive generation of ROS is known to trigger oxidative damage to various biomolecules, such as DNA, proteins and lipids, which in turn may lead to mitochondrial membrane permeabilization [31]. In some cases, ROS may induce both apoptosis and necrosis in tumors. Qu et al. [32] reported GO-induced macrophagic cell death through programmed necrosis in J774A.1 cells and showed that GO toxicity is mediated by activation of toll-like receptor 4 (TLR4) signaling. Macrophage cell death linked to GO exposure was attributed to programmed necrosis mediated by a receptor-interacting protein kinase 1 and 3 complex, downstream of TNF-α induction. Fig. 5a & b are schematic illustrations of the mechanisms by which photosensitizers generate ^1^O_2_, and by which the hybrid of folic acid polyethylene glycol and C_60_ (a spherical fullerene molecule with the formula C_60_ called buckminsterfullerene) conjugated to GO (FA-GO-PEG/C_60_) achieves the combined synergistic effects of photothermal therapy and PDT.

## 4 Summary

In summary, recent studies underpinning the application potential of graphene in the theranostic field have been reviewed. Many groups have utilized graphene in PDT, photothermal therapy and fluorescent imaging for cancer treatment. The combination of imaging and therapy could produce synergistic effects to increase the targeted killing with minimal side effects and with the maintenance of biocompatibility. The scope for functionalization and conjugation of graphene can potentially generate a promising array of theranostic agents. Further in vivo studies will be required to better understand the real-world applications of graphene. Moreover, the aspects of ROS generation, toxicity and potential cancer theranostic approaches for other derivatives of graphene such as graphene nanoribbons, graphene nanoplatelets, three dimensional graphene foams, graphene nanopores, and porous graphene nanosheets will need to be studied. Oxidative stress induced by graphene accumulated in living organs is due to acellular factors such as particle size, particle shape, surface charge, surface functional groups, and light activation, while cellular responses such as mitochondrial respiration, immune cell activation, pH of the medium and physiological redox-regulated functions are critical determinants affecting the production of ROS. To date, the mechanisms and roles of ROS production by most forms of graphene in relation to cancer treatment, are not understood. A basic understanding of graphene-cell interactions (especially ROS generation), as well as the optimal conditions for their proper use, will provide new theranostic platforms in the future.
